# Early prediction of severe morbidity after liver resection using serum albumin and ALBI-score in a mixed patient cohort

**DOI:** 10.1186/s12893-026-04190-7

**Published:** 2026-09-29

**Authors:** Sophie Seiffer, Lars Stellmacher, Aaron Schindler, Uta Pietsch, Thomas C Wiemers, Hans-Jonas Meyer, Robert Sucher, Daniel Seehofer, Hans-Michael Tautenhahn, Uwe Scheuermann

**Affiliations:** 1 Department of Visceral, Transplant, Thoracic and Vascular Surgery, University Hospital Leipzig, Liebigstrasse 20, Leipzig, 04103 Germany; 2 Division of Hepatology, Department of Medicine II, Leipzig University Medical Center, Leipzig, Germany; 3 Department of Anesthesiology and Intensive Care Medicine, University Hospital of Leipzig, Leipzig, Germany; 4 Department of Diagnostic and Interventional Radiology, University Hospital Leipzig, Leipzig, Germany; 5 Department of General-, Visceral- and Transplant Surgery, Medical University of Graz, Graz, Austria

**Keywords:** Liver resection, Outcome, ALBI score, Albumin, Postoperative complication, Morbidity

## Abstract

**Background:**

Early identification of patients at risk for severe morbidity after liver resection (LR) remains challenging. Serum albumin and albumin–bilirubin (ALBI) score reflect liver function, but their prognostic value in the immediate postoperative period is not well defined. This study evaluated perioperative serum albumin and ALBI as early predictors of severe postoperative morbidity in a heterogeneous LR cohort.

**Methods:**

We retrospectively analyzed 150 adult patients undergoing LR at a tertiary center (2016–2018), excluding patients receiving perioperative albumin substitution. Albumin and ALBI were measured preoperatively and on postoperative day 1 (POD1). Associations with intraoperative variables and postoperative outcomes were evaluated. Severe morbidity (Clavien–Dindo ≥ IIIb) within 90 days was the primary endpoint. Predictive performance was evaluated using ROC analysis and logistic regression with bootstrap internal validation.

**Results:**

Severe morbidity occurred in 23 patients (15.3%), including four deaths (2.7%). Albumin and ALBI deteriorated significantly from preoperative values to POD1 (*P* < 0.001) and their relative changes correlated inversely with intraoperative volume substitution (albumin: ρ = −0.380, ALBI: ρ = −0.376; *P* < 0.001 each). On POD1, both parameters demonstrated good discrimination for severe morbidity (AUC 0.776 for albumin; AUC 0.783 for ALBI). Exploratory thresholds for albumin < 29.4 g/L or ALBI > − 1.56 were associated with higher postoperative complication rates (albumin: 66.7% vs. 29.8%, ALBI: 68.8% vs. 30.5%; *P* < 0.001 each) and prolonged hospitalization (albumin: 21.8 vs. 10.1 days, ALBI: 22.3 vs. 10.4 days, *P* < 0.001 each). In multivariate analysis, POD1 albumin (OR 0.859, 95% CI 0.726–0.998; *P* = 0.047) and POD1 ALBI (OR 6.275, 95% CI 1.417–33.684; *P* = 0.015) independently predicted severe morbidity. Adding POD1 ALBI improved the discrimination of both the ACS NSQIP surgical risk calculator and the Breitenstein score (ΔAUC 0.094 and 0.181; *P* = 0.029 and 0.005).

**Conclusions:**

ALBI on POD1 independently predicted severe morbidity after LR and may provide a simple tool for early postoperative risk stratification based on routine laboratory data. External validation is required before routine implementation.

**Supplementary Information:**

The online version contains supplementary material available at https://doi.org/10.1186/s12893-026-04190-7.

## Introduction

Liver resection (LR) is a cornerstone of curative treatment for primary and secondary hepatic malignancies. Despite substantial advances in surgical techniques and perioperative care, LR remains associated with severe postoperative morbidity, which adversely affects recovery, prolongs hospitalization, and increases mortality [1–3].

As postoperative outcomes after LR largely depend on the functional capacity of the liver remnant, assessment of hepatic function is essential for surgical planning and perioperative management [4, 5]. Commonly used scores such as the Child–Pugh and MELD provide a global estimate of hepatic function but poorly predict short-term postoperative risk [6, 7]. Volumetric and functional assessments improve preoperative risk stratification but are resource-intensive and not widely accessible [8, 9]. Moreover, preoperative risk assessment cannot account for intraoperative factors that further affect remnant liver function, including the extent of resection, blood loss, and ischemia time [10, 11]. Early postoperative reassessment of hepatic function is therefore needed to identify patients at increased risk of post-hepatectomy liver dysfunction and subsequent complications.

Serum albumin, reflecting hepatic synthetic capacity and physiological reserve, is a practical predictor of vulnerability. Preoperative hypoalbuminemia independently associates with severe complications and mortality after LR [12, 13], while early postoperative albumin dynamics mirror surgical stress response and correlate with operative magnitude and adverse outcomes [14–16]. Interpretation of postoperative albumin levels, however, is affected by fluid shifts, hemodilution, and perioperative supplementation, potentially limiting their reliability as a standalone marker [17].

The albumin–bilirubin (ALBI) score was originally introduced as an objective measure of liver function in patients with hepatocellular carcinoma (HCC) [18]. By integrating serum albumin and total bilirubin, it reflects both synthetic and excretory hepatic function. Unlike the Child-Pugh score, it relies solely on laboratory values, reducing subjectivity and improving prognostic accuracy [19–21]. Emerging evidence highlights its broader utility for perioperative risk stratification across liver disease, surgery, and systemic stress context [19].

Perioperative data on albumin- and ALBI-based risk stratification after LR remain limited, often focusing on preoperative values in selected cohorts, while the immediate postoperative period is less well characterized. Recent evidence suggests that postoperative ALBI provides strong predictive insight into severe morbidity in patients with HCC [22, 23]. Machine-learning models have further highlighted POD1 ALBI as among the most influential predictors of postoperative complications and mortality [24]. However, its discriminative performance in the early postoperative period after LR, including clinically applicable thresholds and direct comparison with serum albumin, remains insufficiently characterized.

Therefore, we aimed to determine whether preoperative and POD1 serum albumin and ALBI scores can serve as early, reliable predictors of severe morbidity after LR in a real-world, heterogeneous cohort.

## Patients and methods

### Data collection and study population

**Fig. 1:**
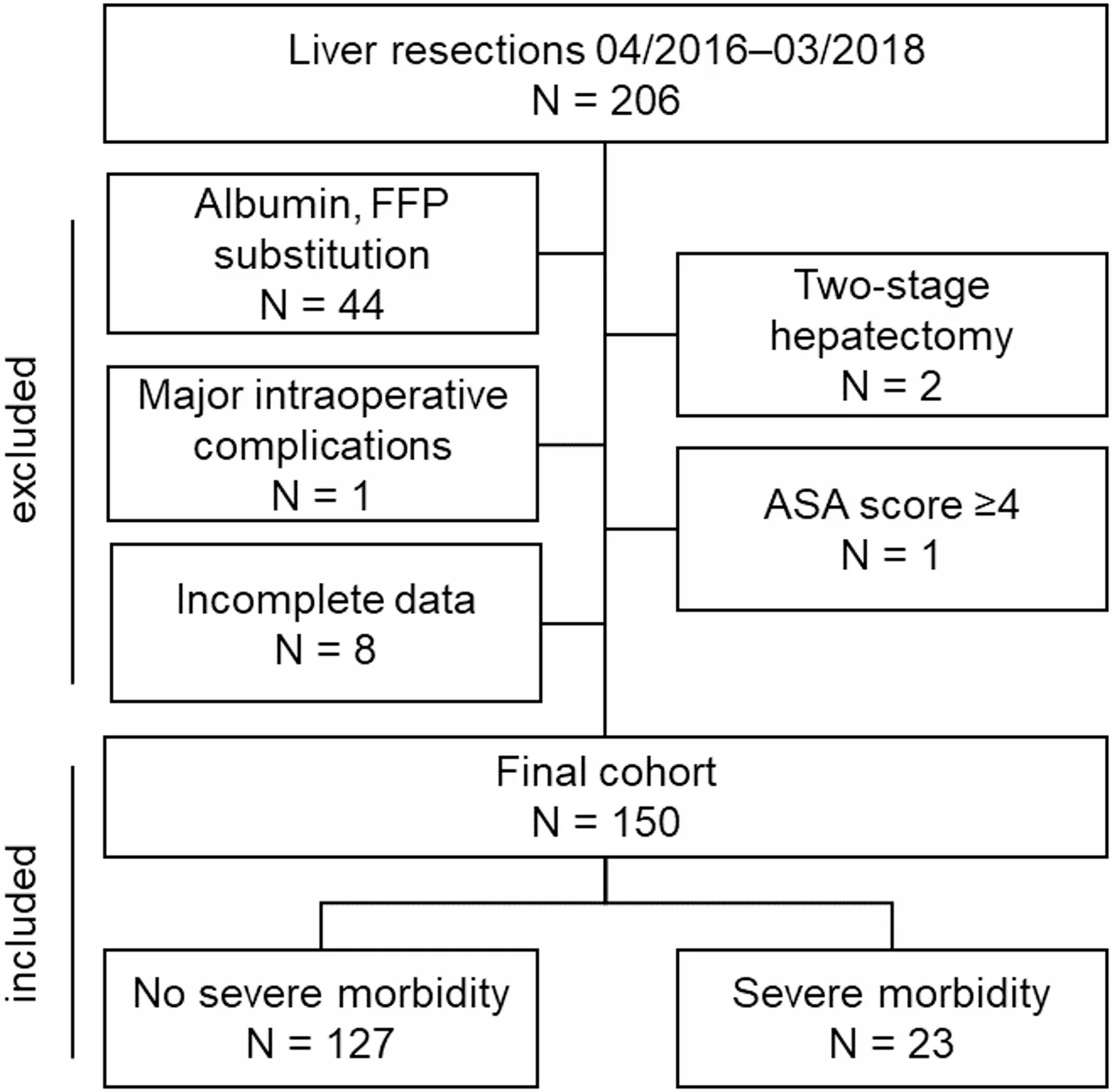
Flowchart of study population selection. Two-stage hepatectomy was performed as in situ split (ALPPS, associating liver partition and portal vein ligation for staged hepatectomy). Severe morbidity was defined as Clavien-Dindo grade ≥ IIIb within 90 days. ASA, American Society of Anesthesiologists; FFP, fresh frozen plasma

Medical records of all adult patients (≥ 18 years) who underwent LR at the University Hospital Leipzig between April 2016 and March 2018 were retrospectively reviewed (*N* = 206). Patients who received perioperative (intraoperatively or on the first postoperative day) albumin substitution (*N* = 44) were excluded to avoid confounding of postoperative albumin measurements. Further exclusions comprised patients with major intraoperative complications (*N* = 1), two-stage surgery (in situ split, *N* = 2), ASA score ≥ IV (*N* = 1) or missing laboratory data for albumin/ALBI analyses (*N* = 8). In situ split combines parenchymal transection with portal vein ligation at the first stage (associating liver partition and portal vein ligation for staged hepatectomy, ALPPS). The final cohort comprised 150 patients. A stratification flow chart is shown in Fig. 1. No imputations were performed for missing data.

Data were extracted from anesthesiology and operative records, including patient history, intraoperative details, histopathological findings, ICU stay, and discharge summaries. The study was approved by the ethics committee of the University of Leipzig (No. 142/18-EK) and conducted in accordance with relevant guidelines; informed consent was waived due to the retrospective design.

All cases were reviewed in a multidisciplinary tumor board. Patients with advanced liver disease, poor general condition, or severe cardiopulmonary comorbidities were generally deemed ineligible for surgery. Portal vein embolization (PVE) with follow-up CT volumetry after approximately four weeks was performed when the anticipated future liver remnant was insufficient, with ≥ 25–30% of total liver volume or ≥ 0.8% of body weight considered adequate for major hepatectomy.

Collected data included demographics (age, gender, BMI), ASA classification, neoadjuvant systemic therapy (type of regimen, individual agents, interval between the last treatment cycle and resection), intraoperative parameters (operative time, blood loss, transfusion, Pringle maneuver, extent of resection, lymphadenectomy), histopathology, and postoperative outcomes.

LRs were classified according to the Brisbane system [25]. Major resections included hemihepatectomy or extended hepatectomy. Procedures involving additional vascular, biliary, or adjacent organ resection were defined as complex resections. Standard lymphadenectomy encompassed lymph nodes of the hepatoduodenal ligament and common hepatic artery.

Parenchymal transection was performed using standard techniques (CUSA for open and ultrasonic shears in laparoscopic resections). Vascular inflow control was applied via Pringle maneuver when indicated. All patients were routinely monitored in the ICU for at least one postoperative night.

Serum albumin and bilirubin levels were measured preoperatively (PRE) and on postoperative day 1 (POD1). All postoperative values were obtained at the routine morning laboratory assessment, 12 to 24 h after the end of surgery; patients without either value in this interval were excluded. The ALBI score was calculated as: ALBI = (log_10_ bilirubin [µmol/L] × 0.66) + (albumin [g/L] × −0.0852) [18]. For each patient, the risk of serious complications according to the ACS NSQIP surgical risk calculator [26] and the Breitenstein score [27] were determined.

### Outcome measures

Postoperative complications within 90 days after LR were recorded, including surgical, cardiopulmonary, renal, thromboembolic, and septic events. Complications were graded according to the Clavien–Dindo (CD) classification, with severe morbidity defined as CD ≥ IIIb [28]. Secondary endpoints included 90-day mortality and length of hospital stay.

### Statistical analysis

Continuous variables are presented as mean ± standard deviation (SD) or median (interquartile range, IQR) for non-normally distributed data. Normality was assessed using the Shapiro–Wilk test. Group comparisons were performed using Student’s t-test, ANOVA, Mann–Whitney U test, Kruskal–Wallis test, Fisher’s exact test, or chi-squared test as appropriate. Receiver operating characteristic (ROC) curves were used to evaluate predictive performance, with optimal cut-offs using Youden’s J statistic [29]. Sensitivity, specificity, predictive values and odds ratios (OR) were additionally calculated at the 10th, 25th, 50th, 75th and 90th percentiles of each marker, the established ALBI grade boundaries [18], and the lower limit of normal for serum albumin. Associations between variables were evaluated using Spearman’s correlation (ρ). Associations with severe morbidity (CD ≥ IIIb) were evaluated using univariate and multivariate logistic regression. Only variables clinically relevant and with *P* < 0.05 in univariate analysis were entered into multivariate analysis. Separate models were fitted for albumin and ALBI; the number of variables included was restricted according to events-per-variable principles to avoid model overfitting. Models were estimated by Firth’s penalized maximum likelihood [30, 31], with confidence intervals (CI) and P values derived from the profile penalized likelihood; collinearity was assessed by variance inflation factors and model stability by bootstrap resampling (2000 replications) with optimism correction. Results are reported as OR with 95% CI. The incremental value of adding POD1 ALBI to the ACS NSQIP calculator and the Breitenstein score was assessed as the increase in the area under the curve (AUC, DeLong test), confirmed by likelihood-ratio tests, and internally validated by bootstrap optimism correction. A two-sided P-value < 0.05 was considered statistically significant. Statistical analyses and figures were generated using SPSS 29.0, R 4.3.3 and GraphPad Prism 10.4.

## Results

### Patient characteristics

A total of 150 patients were included (Table 1). Mean age was 58.8 ± 15.3 years, and 54.7% were male. Most patients were classified as ASA II (54.7%) or ASA III (40.7%). Malignant disease accounted for 72.7% of resections, including primary hepatic malignancies (32.0%) and liver metastases (40.7%). Hepatic steatosis was present in 36.0% and liver cirrhosis in 4.0% of cases; 7.3% underwent preoperative PVE. Neoadjuvant systemic therapy was administered in 36.0% (chemotherapy alone 16.0%, with targeted therapy 18.7%, targeted therapy alone 1.3%). Oxaliplatin was used in 16.7% and irinotecan in 12.0%, with a median interval of 3.0 months (IQR 1.1–9.0) between the last cycle and resection.

**Table 1:** Baseline patient characteristics and surgical procedures

Variables	*N* = 150
Patient characteristics	
Age, years	58.8 ± 15.3
Male (%)	82 (54.7)
BMI, kg/ m^2^	26.4 ± 4.7
ASA score, I/ II/ III (%)	7 (4.7)/ 82 (54.7)/ 61 (40.7)
Diagnosis (%)	
Malignant	109 (72.7)
Primary hepatic, HCC/ CCA/ others	21 (14.0)/ 19 (12.7)/ 8 (5.3)
Metastases, CRLM/ nCRLM	45 (30.0)/ 16 (10.7)
Steatosis hepatis (%)	54 (36.0)
Liver cirrhosis (%)	6 (4.0)
Preoperative PVE (%)	11 (7.3)
Neoadjuvant therapy (%)	54 (36.0)
Surgery	
Reoperation (%)	7 (4.7)
LLR/ OLR (%)	44 (29.3)/ 106 (70.7)
Major resection (%)	52 (34.7)
Type of resection (%)	
Atypical	15 (10.0)
Segmentectomy ± atypical	70 (46.7)
Bisegmentectomy ± atypical	21 (14.0)
Hemihepatectomy left/ right	6 (4.0)/ 10 (6.7)
Extended hemihepatectomy left/ right	7 (4.7)/ 21 (14.0)
Pringle maneuver (%)	97 (64.7)
PM duration, minutes	29 ± 12.2
Operation time, minutes	306 ± 104

*ASA* American Society of Anesthesiologists, *BMI* body mass index, *CCA* Cholangiocarcinoma, *CRLM* Colorectal liver metastases, *HCC* Hepatocellular carcinoma, *LLR* Laparoscopic liver resection, *nCRLM* non-colorectal liver metastases, *OLR* Open liver resection, *PM* Pringle maneuver, *PVE* Portal vein embolization

Open LR was performed in 106 cases (70.7%), and 52 (34.7%) were major resections. The Pringle maneuver was used in 64.7%, with a mean duration of 29 ± 12.2 min. Mean operative time was 306 ± 104 min. Lymphadenectomy was performed in 37.3%, biliodigestive anastomosis in 7.3%, and 16.0% of procedures were classified as complex.

Median intraoperative blood loss was 300 mL (IQR 200–560 mL; *N* = 128), and median intraoperative fluid administration was 3,000 mL (IQR 2,000–4,000 mL; *N* = 149).

### Perioperative albumin level and ALBI Score

**Fig. 2:**
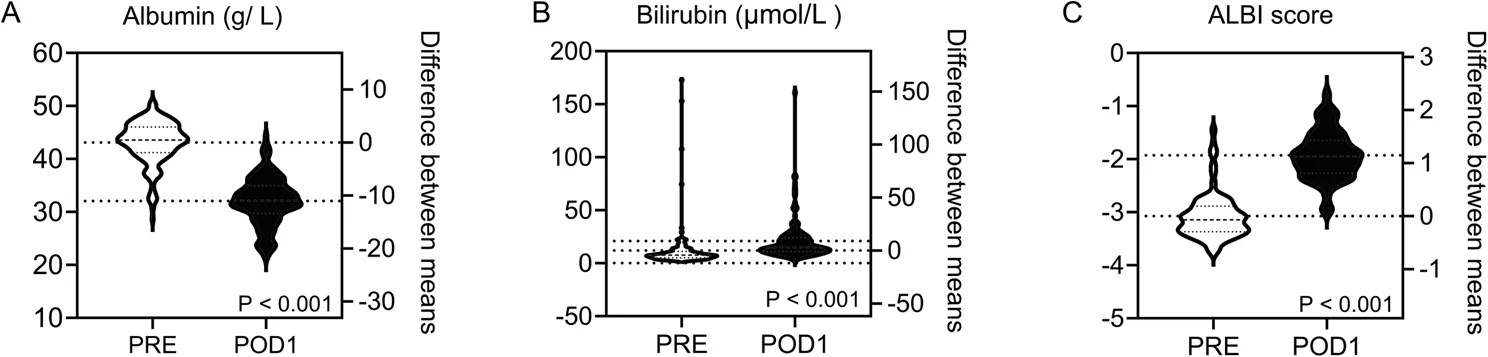
Violin plots showing the distribution of (**A**) serum albumin, (**B**) bilirubin concentrations and (**C**) ALBI scores measured preoperatively and on postoperative day 1 in 150 patients undergoing liver resection. Dotted horizontal lines indicate mean values. The right y-axis denotes the difference between PRE and POD1 means. P-values were calculated using paired t-tests. ALBI, albumin–bilirubin score; POD1, postoperative day 1; PRE, preoperative

Mean serum albumin decreased from 43.1 ± 4.0 g/L preoperatively to 32.1 ± 4.5 g/L on POD1 (− 25%), bilirubin increased from 12.1 ± 20.8 µmol/L to 20.9 ± 18.8 µmol/L, and the ALBI score increased from − 3.07 ± 0.40 to − 1.93 ± 0.50 (*P* < 0.001 each) (Fig. 2). Preoperatively, decreased albumin levels (below the reference value of 35 g/L) were observed in eight patients (5.3%), while elevated bilirubin levels (above the reference value of 21 µmol/L) were found in 12 patients (8.0%).

**Fig. 3:**
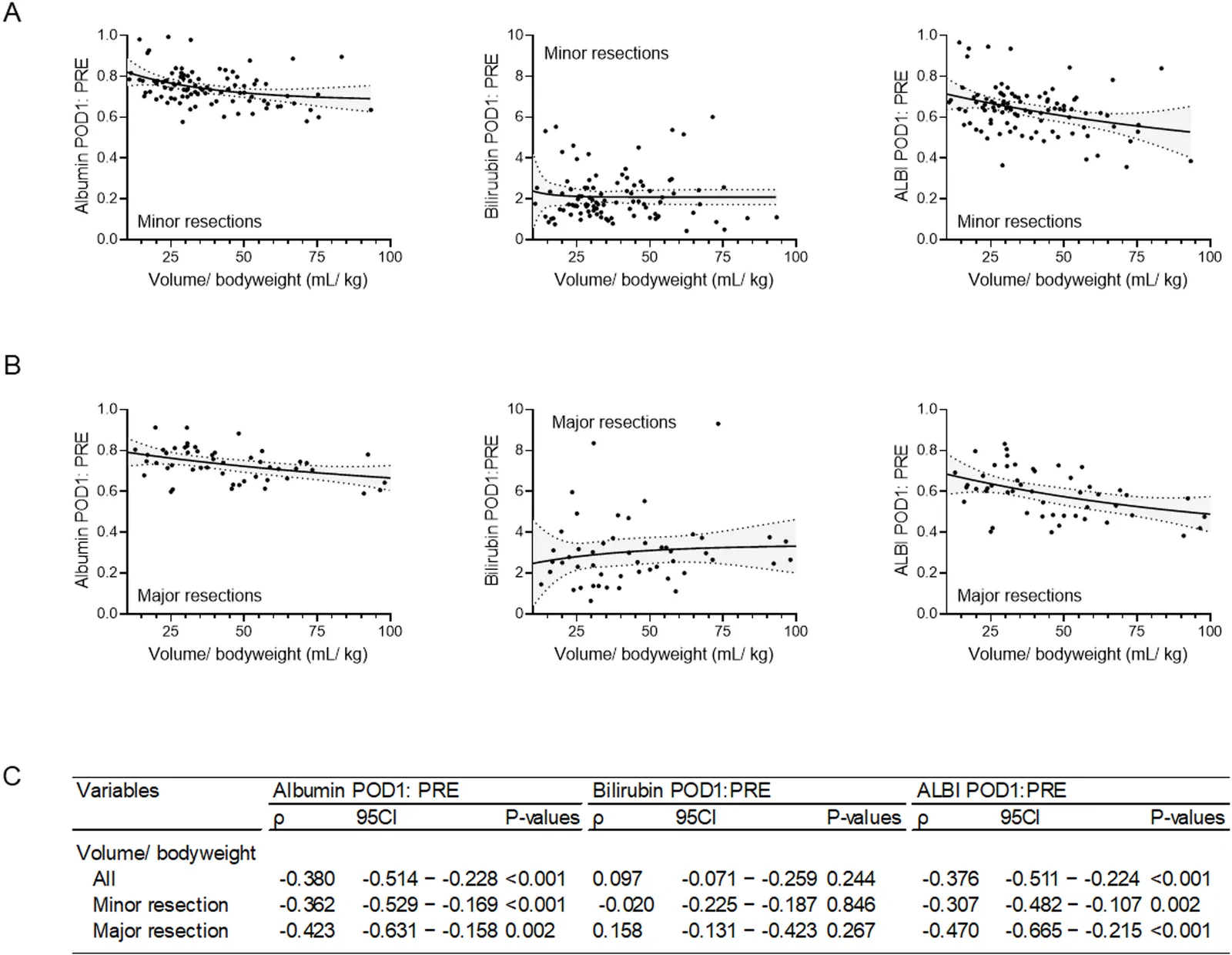
Correlation of the relative perioperative change (POD1:PRE ratio) in albumin, bilirubin and ALBI with intraoperative volume substitution per body weight in (**A**) minor and (**B**) major liver resections. **C** Spearman’s correlation analysis between intraoperative volume substitution and POD1:PRE ratios for serum albumin, bilirubin and ALBI. P-values were derived from two-sided rank-based tests of the correlation coefficients against zero. ALBI, albumin–bilirubin score; CI, confidence interval; POD1, postoperative day 1; PRE, preoperative

Intraoperative volume substitution adjusted for body weight was significantly associated with relative changes from preoperative values to POD1 in serum albumin levels and ALBI scores (albumin: ρ = −0.380; ALBI: ρ = −0.376; both *P* < 0.001). These associations were observed in both minor and major resections (Fig. 3). In an exploratory subgroup analysis of patients with available blood loss data (*N* = 128), intraoperative blood loss was modestly correlated with POD1 albumin (ρ = −0.234, *P* = 0.008), POD1 bilirubin (ρ = 0.194, *P* = 0.028) and POD1 ALBI score (ρ = 0.267, *P* = 0.002).

### Prediction of severe postoperative morbidity

**Fig. 4:**
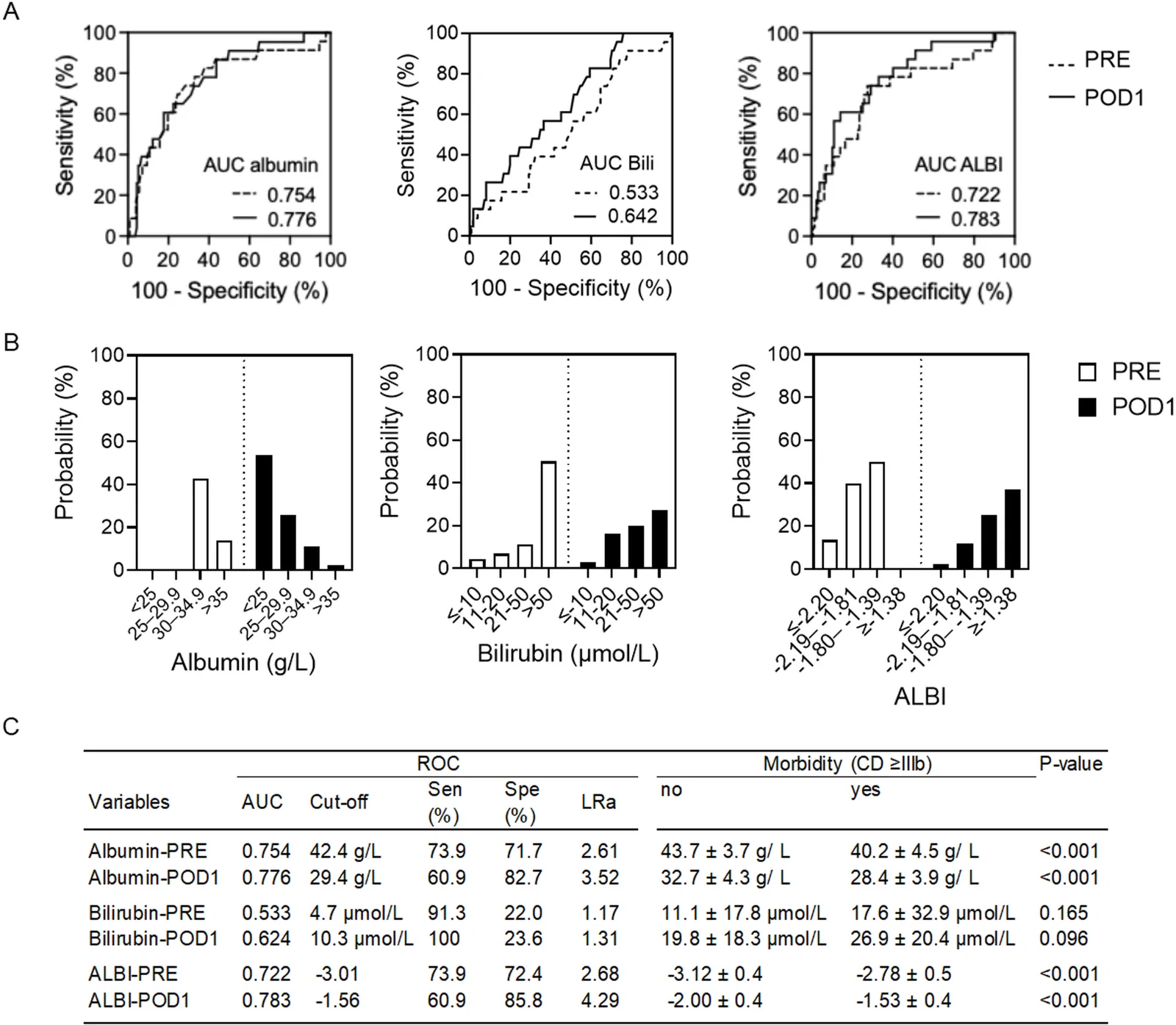
Receiver operating characteristic (ROC) curves and predicted probabilities of perioperative serum albumin and albumin–bilirubin (ALBI) scores for severe postoperative morbidity (Clavien–Dindo grade ≥ IIIb) in 150 patients undergoing liver resection. **A** ROC curves comparing the discriminatory performance of preoperative (PRE) and postoperative day 1 (POD1) serum albumin, bilirubin and the ALBI score. **B** Probability of severe postoperative morbidity according to serum albumin, bilirubin concentration and ALBI score. **C** Area under the ROC curve (AUC), calculated cut-off values, sensitivity, specificity, and likelihood ratios of severe postoperative complications, along with mean marker values in patients with and without severe morbidity. Cut-off values were derived from ROC analyses. Sensitivity (Sen) and specificity (Spe) are reported at the indicated cut-offs. ALBI, albumin–bilirubin score; AUC, area under the curve; CD, Clavien–Dindo; LRa, likelihood ratio; POD1, postoperative day 1; PRE, preoperative; ROC, receiver operator characteristic; Sen, Sensitivity; Spe, specificity

Overall morbidity occurred in 58 patients (38.7%), with major complications (CD ≥ IIIb) in 23 (15.3%), including four deaths (2.7%). All 23 events became clinically manifest after POD1 sampling (one later the same day, the others between POD2 and POD17); 11 had occurred by POD3. ROC analysis demonstrated good discrimination of albumin and ALBI for severe morbidity preoperatively and on POD1, with the highest AUC observed for POD1 values (0.776 and 0.783, respectively) (Fig. 4A). At the ROC-derived POD1 cut-offs of 29.4 g/L for albumin and − 1.56 for ALBI, both markers demonstrated equal sensitivity (60.9%), while ALBI showed higher specificity (85.8% vs. 82.7%) and a higher positive likelihood ratio (4.29 vs. 3.52) (Fig. 4C). Negative predictive values exceeded 92% for both markers (albumin: 92.1%; ALBI: 92.4%).

Due to the limited predictive value of bilirubin for severe postoperative morbidity, subsequent analyses focused on serum albumin and the ALBI score.

Patients with POD1 albumin < 29.4 g/L or ALBI > − 1.56 more frequently underwent major resection (47.2% vs. 30.7%, *P* = 0.069; 56.3% vs. 28.8%, *P* = 0.004), and open surgery (94.4% vs. 63.2%, *P* < 0.001; 93.8% vs. 64.4%, *P* = 0.001), and received greater intraoperative volume substitution (55 vs. 36 mL/kg; 56 vs. 37 mL/kg; both *P* < 0.001). They exhibited higher overall complication rates (66.7% vs. 29.8%; 68.8% vs. 30.5%; both *P* < 0.001) and prolonged hospitalization (21.8 vs. 10.1 days; 22.3 vs. 10.4 days; both *P* < 0.001) (Table 2), with all deaths occurring in high-risk patients defined by these cut-offs.

**Table 2:** Association of POD1 albumin and ALBI levels with patient characteristics, surgical factors, and postoperative outcomes based on predefined ROC-derived cut-off values

Variables	Total	Albumin-POD1, g/ L		*P*-value	ALBI-POD1		*P*-value
		> 29.4	< 29.4		< -1.56	> -1.56	
	*N* = 150	*N* = 114	*N* = 36		*N* = 118	*N* = 32	
Patient characteristics							
Age, years	58.8 ± 15.3	57.9 ± 15.1	61.7 ± 15.9	0.191	58.1 ± 15.3	61.4 ± 15.3	0.278
Male (%)	82 (54.7)	66 (57.9)	16 (44.4)	0.158	66 (55.9)	16 (50.0)	0.550
BMI, kg/ m^2^	26.4 ± 4.7	26.9 ± 4.8	24.7 ± 3.9	0.015	26.8 ± 4.8	24.9 ± 4.3	0.045
ASA score III (%)	61 (40.7)	43 (37.7)	18 (50.0)	0.191	44 (37.3)	17 (53.1)	0.106
Malignant tumor (%)	109 (72.7)	80 (70.2)	29 (80.6)	0.223	81 (68.6)	28 (87.5)	0.034
Steatosis hepatis (%)	54 (36.0)	44 (38.6)	10 (27.8)	0.366	43 (36.4)	11 (34.4)	0.906
Liver cirrhosis (%)	6 (4.0)	6 (5.3)	0	0.160	6 (5.1)	0	0.193
Neoadjuvant therapy (%)	54 (36.0)	38 (33.3)	16 (44.4)	0.226	39 (33.1)	15 (46.9)	0.148
Surgery							
OLR (%)	106 (70.7)	72 (63.2)	34 (94.4)	< 0.001	76 (64.4)	30 (93.8)	0.001
Major resection (%)	52 (34.7)	35 (30.7)	17 (47.2)	0.069	34 (28.8)	18 (56.3)	0.004
Pringle maneuver (%)	97 (64.7)	74 (64.9)	23 (63.9)	0.911	77 (65.3)	20 (62.5)	0.773
PM duration, minutes	29 ± 12.2	30 ± 12.3	27 ± 11.9	0.372	30 ± 12.3	28 ± 12.0	0.582
Volume/ bodyweight, mL/ kg	41 ± 20.1	36 ± 16.9	55 ± 23.3	< 0.001	37 ± 17.1	56 ± 23.6	< 0.001
Operation time, minutes	306 ± 104	296 ± 101	340 ± 107	0.024	299 ± 104	332 ± 102	0.112
Morbidity (%)							
Overall complications	58 (38.7)	34 (29.8)	24 (66.7)	< 0.001	36 (30.5)	22 (68.8)	< 0.001
Clavien-Dindo ≥IIIb	23 (15.3)	9 (7.9)	14 (38.9)	< 0.001	9 (7.6)	14 (43.8)	< 0.001
Ascites or pleural effusion	12 (8.0)	5 (4.4)	7 (19.4)	0.004	7 (5.9)	5 (15.6)	0.073
Delayed wound healing	28 (18.7)	17 (14.9)	11 (30.6)	0.036	18 (15.3)	10 (31.3)	0.039
Renal failure	8 (5.3)	4 (3.5)	4 (11.1)	0.077	3 (2.5)	5 (15.6)	0.003
Hospital stay, days	13.0 ± 9.4	10.1 ± 5.9	21.8 ± 12.6	< 0.001	10.4 ± 6.0	22.3 ± 13.4	< 0.001

*ALBI* Albumin–bilirubin score, *ASA* American Society of Anesthesiologists, *BMI* Body mass index, *OLR* Open liver resection, *PM* Pringle maneuver, *POD1* postoperative day 1

Absolute perioperative changes (PRE–POD1) in albumin and ALBI were not predictive for severe morbidity. Relative change metrics correlated strongly with POD1 values and therefore were not retained as separate predictors.

POD1 albumin and ALBI differed significantly by operative approach and extent: open LR was associated with lower POD1 albumin than laparoscopic LR (31.35 vs. 34.65 g/L; *P* < 0.001) and worse POD1 ALBI (− 1.85 vs. −2.31; *P* < 0.001). Major LR also showed lower POD1 albumin than minor LR (31.10 vs. 32.80 g/L; *P* = 0.027) and worse POD1 ALBI (− 1.83 vs. −2.03; *P* = 0.007). The perioperative decline in albumin (PRE–POD1) was greater following open compared with laparoscopic resections (12.05 vs. 9.80 g/L; *P* < 0.001). Neither POD1 albumin nor POD1 ALBI differed between patients with and without neoadjuvant systemic therapy (31.7 vs. 32.3 g/L, *P* = 0.422; −1.87 vs. −1.96, *P* = 0.239).

Predictive performance was additionally evaluated across a range of alternative cut-off values (Supplementary Tables S1A and S1B). For POD1 ALBI, severe morbidity was significantly associated with all but the two lowest thresholds examined (OR 3.84–9.42). Sensitivity ranged from 26.1% to 100% and specificity from 7.1% to 92.9%. For POD1 albumin, the association was significant at four of the five thresholds examined (OR 7.42–10.76), with sensitivity ranging from 34.8% to 100% and specificity from 11.8% to 95.3%. Based on established ALBI grade thresholds [18] (grade 1 ≤ − 2.60; grade 2 > − 2.60 to ≤ − 1.39; grade 3 > − 1.39), POD1 ALBI grade distribution was: grade 1, *N* = 9; grade 2, *N* = 121; grade 3, *N* = 20. Rates of severe morbidity increased progressively with higher ALBI grade (0%, 13.2%, and 35.0%, respectively; *P* = 0.005), and three of the four deaths occurred in grade 3.

### Multivariate analysis

In univariate analysis, ASA score, OLR, extent of LR, intraoperative volume substitution per body weight, and preoperative and POD1 serum albumin and ALBI scores were significantly associated with severe morbidity. In multivariate analysis, POD1 albumin (OR 0.859, 95% CI 0.726–0.998; *P* = 0.047; Table 3) and POD1 ALBI (OR 6.275, 95% CI 1.417–33.684; *P* = 0.015; Table 4) remained independently associated with severe morbidity, whereas the preoperative values did not achieve statistical significance. No variance inflation factor exceeded 1.84, and both models were robust in bootstrap internal validation, with optimism-corrected AUC of 0.808 in the albumin model and 0.805 in the ALBI model (Supplementary Tables S2A and S2B). The ASA score retained an independent association in both models.

**Table 3:** Predictors of severe postoperative morbidity (Clavien–Dindo grade ≥ IIIb) in 150 patients who underwent liver resection, assessed in separate multivariate models for albumin

Variables	Morbidity (Clavien-Dindo ≥IIIb)			
	UVA	Multivariate analysis		
	P-value	OR	95% CI	P-value
Patient characteristic				
ASA score I-II vs. III	0.001	3.514	1.268–10.669	0.015
Laboratory parameters				
Albumin-PRE	< 0.001	0.962	0.831–1.123	0.611
Albumin-POD1	< 0.001	0.859	0.726–0.998	0.047
Surgery				
OLR vs. LLR	0.031	0.650	0.117–2.536	0.555
Minor vs. major LR	0.020	2.240	0.831–6.137	0.110

Intraoperative volume substitution per body weight was significant in univariate analysis (*P* = 0.014) but not included in the multivariate models in accordance with the events-per-variable restriction (see Methods). The following variables were tested in the univariate analysis but failed to show significance: patient age, gender, and body mass index, hepatic steatosis, cirrhosis, neoadjuvant systemic therapy, preoperative portal vein embolization, malignant disease, preoperative and POD1 serum bilirubin, duration of operation, Pringle maneuver, lymphadenectomy, biliodigestive anastomosis, intraoperative blood loss, complex resections

*ASA* American Society of Anesthesiologists, *CI* confidence interval, *LLR* laparoscopic liver resection, *minor* *LR* minor liver resection, *major* *LR* major liver resection, *OLR* open liver resection, *OR* odds ratio, *PRE* preoperative, *POD1* postoperative day 1, *UVA* univariate analysis

**Table 4:** Predictors of severe postoperative morbidity (Clavien–Dindo grade ≥ IIIb) in 150patients who underwent liver resection, assessed in separate multivariate models for ALBI

Variables	Morbidity (Clavien-Dindo ≥IIIb)			
	UVA	Multivariate analysis		
	P-value	OR	95% CI	P-value
Patient characteristic				
ASA score I-II vs. III	0.001	3.777	1.368–11.493	0.010
Laboratory parameters				
ALBI-PRE	0.001	1.095	0.271–3.876	0.893
ALBI-POD1	< 0.001	6.275	1.417–33.684	0.015
Surgery				
OLR vs. LLR	0.031	0.626	0.115–2.394	0.514
Minor vs. major LR	0.020	1.998	0.730–5.529	0.176

Intraoperative volume substitution per body weight was significant in univariate analysis (*P* = 0.014) but not included in the multivariate models in accordance with the events-per-variable restriction (see Methods). The following variables were tested in the univariate analysis but failed to show significance: patient age, gender, and body mass index, hepatic steatosis, cirrhosis, neoadjuvant systemic therapy, preoperative portal vein embolization, malignant disease, preoperative and POD1 serum bilirubin, duration of operation, Pringle maneuver, lymphadenectomy, biliodigestive anastomosis, intraoperative blood loss, complex resections

*ALBI* albumin–bilirubin score, *ASA* American Society of Anesthesiologists, *CI* confidence interval, *LLR* laparoscopic liver resection, *minor LR* minor liver resection, *major LR* major liver resection, *OLR* open liver resection, *OR* odds ratio, *PRE* preoperative, *POD1* postoperative day 1, *UVA* univariate analysis

### Incremental value of POD1 ALBI over preoperative risk models

POD1 ALBI alone reached an AUC of 0.783 for severe morbidity, compared with 0.701 (ACS NSQIP surgical risk calculator) and 0.601 (Breitenstein score). Its addition raised the AUCs to 0.795 (NSQIP calculator; ΔAUC 0.094, *P* = 0.029) and 0.782 (Breitenstein score; ΔAUC 0.181, *P* = 0.005); likelihood-ratio tests confirmed its contribution in both models (each *P* < 0.001; Supplementary Table S3).

## Discussion

In this retrospective analysis of 150 patients undergoing LR, POD1 serum albumin and ALBI showed comparable discrimination for severe morbidity (AUC 0.776 and 0.783, respectively), and both retained independent associations in multivariate models. Patients with POD1 ALBI values above the identified threshold of − 1.56 had substantially higher complication rates and prolonged hospitalization.

Initially developed as an objective measure of hepatic function in patients with HCC, the ALBI score has since evolved into a widely adopted, laboratory-based index that is highly sensitive to subtle deterioration in liver function. Its utility has been demonstrated across the spectrum of chronic liver disease and in broader clinical contexts [18–21]. However, most prior studies have focused on patients with underlying chronic liver disease, in whom baseline hepatic impairment is a major determinant of postoperative risk [32]. The present study extends the application of ALBI to the immediate postoperative period, specifically within a heterogeneous cohort predominantly characterized by non-cirrhotic liver parenchyma.

Several studies have evaluated ALBI for risk assessment in liver surgery. The majority examined the score preoperatively, addressing whether pre-existing hepatic functional reserve determines subsequent outcome. Alone or combined with APRI, preoperative ALBI predicted post-hepatectomy liver failure (PHLF), morbidity and mortality [20, 33–38], outperforming the Child–Pugh classification [20] and the MELD score [38] in direct comparisons. In our cohort, serum albumin and ALBI were likewise associated with severe morbidity both preoperatively and on POD1. In multivariate analysis, however, only the POD1 values retained an independent association, which may reflect their integration of baseline hepatic reserve with the early physiological response to the operative insult.

Other models incorporating postoperative ALBI have recently been proposed for patients with HCC [22–24]. Akabane et al. [22] grouped ALBI trajectories up to POD5 into risk clusters that outperformed the preoperative score alone. Another study found a composite index of perioperative change in ALBI and platelet count independently associated with severe complications [23]. Consistently, POD1 and POD3 ALBI have been identified as the strongest predictors of severe morbidity among a range of pre- and postoperative variables in a machine-learning model [24]. The prognostic signal of early postoperative ALBI thus proved robust across analytical approaches. Direct comparisons of discrimination are nevertheless limited by differences in case mix, event rates, endpoint definitions and validation strategies, and none of these models has yet been evaluated in indications for resection other than HCC or undergone external validation.

While albumin is a well-established prognostic marker in surgical patients, its long half-life of approximately 20 days implies that early postoperative levels primarily reflect baseline hepatic function and perioperative fluid shifts rather than acute changes in hepatic synthetic capacity [14–16, 39]. The early postoperative decline in albumin has therefore been described as a marker of surgical stress that correlates with the extent of surgery [14, 40]. This is reflected by the inverse correlation between POD1 albumin and intraoperative volume substitution observed in our study. By contrast, bilirubin kinetics are considerably faster, and early postoperative elevations more likely reflect hepatocellular or biliary injury—such as transient impairment of biliary excretion and microcirculatory alterations in the remnant liver—rather than reduced synthetic function. Consistently, ischemia time showed a weak association with POD1 bilirubin but not with POD1 albumin among patients who underwent a Pringle maneuver in our study (data not shown). Recent evidence further supports the early prognostic relevance of bilirubin, demonstrating significant elevations from POD1 onwards in patients who develop PHLF [41, 42]. By integrating albumin and bilirubin, ALBI captures both synthetic and excretory liver function and may thus provide a more liver-specific assessment of postoperative hepatic impairment than albumin alone [19]. In a single POD1 value, however, the transient effects of surgical stress and fluid shifts are inseparable from incipient impairment of remnant function.

In our cohort, POD1 ALBI predicted severe morbidity independently of ASA class, surgical approach, and extent of resection (Table 3), indicating an initial functional vulnerability of the remnant liver that does not simply correspond to baseline risk or operative magnitude. In line with this, POD1 ALBI provided incremental prognostic information over the ACS NSQIP surgical risk calculator and the Breitenstein score, both of which estimate risk from multiple preoperative variables. A single POD1 value showed numerically higher discrimination for severe morbidity than either model, significantly improved both when added (Supplementary Table S3), and may thus refine preoperative risk assessment with the immediate postoperative response.

We further tested which threshold might be useful to identify patients at increased risk of severe morbidity. Among the cut-offs examined, − 1.56 provided the most favorable balance between sensitivity and specificity, although it remains exploratory and requires confirmation in independent cohorts. This cut-off lies within ALBI grade 2 and close to the grade 2/3 threshold of the original classification by Johnson et al. [18]. Applying this classification, no patient in grade 1 developed severe morbidity, whereas rates rose to 13.2% in grade 2 and 35.0% in grade 3 (*P* = 0.005), with nearly fourfold odds above the grade 2/3 boundary (OR 3.84, 95% CI 1.33–11.04). The association likewise persisted across alternative cut-offs spanning the observed range of POD1 ALBI values (Supplementary Table S1B). The choice of cut-off therefore affected accuracy, while the association itself held throughout. Across the entire range of cut-offs examined, the negative predictive value remained above 87% and exceeded 92% at the ROC-derived cut-off. Against the baseline risk of 15.3%, a favorable value thus approximately halved the residual risk of severe morbidity to 7.6% without excluding it. Importantly, POD1 ALBI relies solely on routine laboratory parameters and requires no additional testing, costs, or patient burden.

In clinical practice, POD1 ALBI may therefore inform decisions at a stage when management remains modifiable. A favorable value in an otherwise stable patient supports standard enhanced recovery pathways, including routine ward transfer and conventional discharge planning. An impaired value may represent either a transient response to the operative insult or evolving postoperative deterioration and should therefore prompt reassessment within the overall clinical course. Initial measures include review by an experienced hepatobiliary team, intensified laboratory surveillance, and adjustment of potentially hepatotoxic or nephrotoxic medication. If the score deteriorates or additional warning signs develop, escalated monitoring and early cross-sectional imaging may be appropriate to identify reversible causes such as biliary leakage, vascular compromise, or intra-abdominal infection, which require timely treatment. In either situation, POD1 ALBI adds an early objective parameter for risk stratification without replacing the overall assessment of the patient; neither de-escalation nor escalation of care should follow from the score alone. Recently proposed models based on serial ALBI values become applicable from POD3 at the earliest [22–24], and the ISGLS definition of PHLF applies from POD5 onwards [4]. In the present cohort, however, nearly half of all severe complications (11 of 23) had become clinically manifest by POD3. Our study demonstrates that prognostic information is already available in this early decision gap, from a single measurement on the first postoperative day.

### Limitations

Several limitations should be acknowledged. The retrospective, single-center design may limit the generalizability and introduce potential selection bias. The exclusion of patients receiving perioperative albumin substitution, while necessary to avoid confounding of postoperative albumin measurements, likely removed a higher-risk subgroup and may have led to an underestimation of overall morbidity. The relatively low number of severe morbidity events constrained multivariate modeling and may affect model stability. Similarly, the PVE subgroup (*N* = 11) was too small for reliable analysis; whether regeneration relates to postoperative ALBI or morbidity remains a question for prospective study. ROC-derived cut-offs are data-driven and exploratory; as they maximize sensitivity and specificity within the derivation cohort, their apparent accuracy is optimistic. In addition, the predictive values observed here depend on the prevalence of severe morbidity and may vary with case mix. Prospective external validation is therefore required before clinical implementation [29, 43, 44]. Moreover, the extent to which the early postoperative ALBI change reflects surgical stress versus incipient remnant liver dysfunction cannot be determined from the present data. Finally, only POD1 measurements were analyzed; serial assessments could provide additional insights into postoperative functional dynamics [22, 24].

## Conclusion

Impaired POD1 ALBI was associated with higher rates of overall complications, severe morbidity, and prolonged hospitalization, supporting its role as a clinically meaningful risk stratification tool after LR. POD1 ALBI provided independent predictive information beyond baseline risk (e.g., ASA) and operative complexity. Unlike albumin alone, it integrates the sensitivity of bilirubin to acute postoperative hepatic injury and may thus also capture incipient impairment of remnant function.

This study is novel in focusing on the immediate postoperative period (POD1) and a stringent endpoint (CD ≥ IIIb within 90 days) in a heterogeneous liver resection cohort, while excluding patients receiving perioperative albumin to minimize biomarker confounding. By relying solely on routine laboratory values, POD1 ALBI may serve as a low-barrier tool to guide intensified surveillance and earlier diagnostic or therapeutic interventions within the overall clinical course. External validation and incorporation into multimodal risk models combining functional, volumetric, and clinical parameters are required before routine implementation.

## Supplementary Information

Supplementary Material 1.

Supplementary Material 2.

Supplementary Material 3.

## Data Availability

Our database contains highly sensible data which may provide insight in clinical and personnel information about our patients and lead to identi cation of these patients. Therefore, according to organizational restrictions and regulations these data cannot be made publicly available. However, the datasets used and/or analyzed during the current study are available from the corresponding author on reasonable request.

## Abbreviations

ACS NSQIP: American College of Surgeons National Surgical Quality Improvement Program
ALBI: Albumin–bilirubin score
ALPPS: Associating liver partition and portal vein ligation for staged hepatectomy
APRI: Aspartate aminotransferase-to-platelet ratio index
ASA: American Society of Anesthesiologists
BMI: Body mass index
CD: Clavien–Dindo classification
ICU: Intensive care unit
ISGLS: International Study Group of Liver Surgery
LLR: Laparoscopic liver resection
MELD: Model for End-Stage Liver Disease
OLR: Open liver resection
PHLF: Post-hepatectomy liver failure
POD1: Postoperative day 1
POD3: Postoperative day 3
POD5: Postoperative day 5
PRE: Preoperative
ROC: Receiver operating characteristic
SD: Standard deviation
